# A vision foundation model approach to quantitative bone marrow fibrosis assessment

**DOI:** 10.1016/j.jpi.2026.100713

**Published:** 2026-08-26

**Authors:** Justin Johnson, Mohamed E. Salama

**Affiliations:** a New York University Grossman School of Medicine, Department of Pathology, New York City, NY, United States of America; b Sonic Healthcare USA, Dallas, TX, United States of America; c University of Texas, San Antonio, Department of Pathology and Laboratory Medicine, San Antonio, TX, United States of America

**Keywords:** Bone marrow fibrosis, Reticulin, Computational pathology, Multiple instance learning, UNI2-h, Merz grid, Myeloproliferative neoplasms

## Abstract

**Background:**

Bone marrow reticulin fibrosis is prognostic in myeloproliferative neoplasms (MPNs). However, fibrosis assessment is limited by interobserver variability. Computational approaches address this, but remain anchored to qualitative assessment, have limited scalability, and none have explicitly evaluated marrow adiposity. Here we developed a pipeline to predict fibrosis scores using vision-transformer (ViT) models.

**Methods:**

Slide from 643 bone marrow cores from 391 patients with a MPN were digitized and analyzed. Cores were tiled, and Merz grids were overlaid on whole-slide images. Fiber-grid intersections were automatically counted, and ViT models were developed using grid scores as labels. Fat-excluded models were also developed, and sampling strategies were swept. All-length score reflects reticulin intersections relative to all eligible marrow-core grid length, whereas the fat-excluded score reflects reticulin intersections relative to the non-fat eligible marrow compartment. Performance was tested on an unseen patient cohort.

**Results:**

Systematic-random sampling of 64 (SR64) tiles per slide had the strongest validation performance across sampling methods, whereas random sampling of 16 tiles showed the strongest correlation with the prior validated manual Merz-grid method. On testing, the SR64 all-length model achieved root mean squared error (RMSE) 2.97, *R*^2^ 0.86, and Pearson *r* 0.93. Meanwhile, SR64 fat-exclusion resulted in RMSE 2.47, *R*^2^ 0.87, and Pearson *r* 0.93. Compared with manual Merz-grid measurements, all-length predictions showed stronger correlation (Pearson *r* = 0.745) than fat-excluded predictions (*r* = 0.439) and automated all-length Merz counting (*r* = 0.687). Cluster visualization demonstrated plausible tile phenotypes associated with model predictions.

**Conclusions:**

ViT-based models can predict automated Merz-derived bone marrow fibrosis. The all-length model strongly correlated with the prior validated manual Merz-grid method. Meanwhile, limited tile sampling preserved strong performance. These findings support a scalable and reproducible framework for fibrosis quantification in myeloproliferative neoplasms.

## Highlights

• Whole-slide image UNI2-h multiple instance learning models predict continuous marrow fibrosis scores.
• Limiting tile sampling preserves model performance, improving computational efficiency.
• Cluster-based SHAP links model predictions to biologically plausible marrow phenotypes.

## Introduction

Bone marrow fibrosis is an important morphological and prognostic feature in patients with myeloproliferative neoplasms (MPNs). Reticulin fibrosis is commonly graded semi-quantitatively using the revised European Consensus 0–3 scale; however, manual grading is limited by poor interobserver reproducibility and inability to monitor disease progression or response quantitatively. These limitations create an opportunity for computational pathology approaches that may support more reproducible fibrosis assessment and future outcome-based studies.

Prior work has demonstrated the feasibility of computational assessment of bone marrow fibrosis, including continuous fibrosis quantification and machine learning–based fibrosis indexing in MPNs. Ryou et al. developed the Continuous Indexing of Fibrosis framework, which used pairwise image comparisons to assign reticulin-stained marrow regions a continuous fibrosis score from 0 to 1 and achieved approximately 94% accuracy in identifying the higher-fibrosis member of an image pair.^1^ The same group subsequently transferred this approach to hematoxylin and eosin (H&E)-stained sections, achieving approximately 93% pairwise/ranking accuracy relative to reticulin-derived fibrosis indexing.^2^ In 2025, D'Abbronzo et al. developed an automated artificial intelligence (AI) tool for reticulin fibrosis grading using an InceptionV3-based model on whole-slide images (WSIs), achieving a Cohen's kappa of 0.831 against expert hematopathologists.^3^ Yu et al. developed a quantitative bone marrow pathology analysis platform for MPNs using U2-Net and ResNet-based models, achieving approximately 0.9 accuracy for fibrosis grading.^4^ More recently, Srisuwananukorn et al. used AI to differentiate prefibrotic primary myelofibrosis from essential thrombocythemia and found that marrow adiposity was a distinguishing morphological feature.^5^

Despite these advances, existing approaches remain limited, to various degrees, by reliance on observer-defined regions, pairwise relative ranking, categorical scoring, or semi-quantitative pathologist-derived fibrosis labels. Manual or region-based sampling may also limit scalability and incompletely capture the spatial heterogeneity of reticulin fibrosis across WSIs. In addition, although marrow adiposity has emerged as a potentially informative feature in MPN classification, prior fibrosis models have not explicitly evaluated the impact of adiposity on fibrosis scoring or compared fat- and non-normalized quantitative fibrosis measures.

In this study, we developed a continuous, slide-level fibrosis prediction framework using whole-slide reticulin images. All slide annotations were generated using a segmentation model and retained marrow regions were tiled and analyzed using linear length density approximation based on stereological principles. The fibrosis quantification methodology was adapted from stereology-based reticulin assessment described by Salama et al., which used systematic uniform random sampling and manual Merz-grid line counting to estimate reticulin fiber length density in bone marrow biopsies.^6^ In that study, stereology-based assessment was associated with average manual pathologist scoring with an *R*^2^ of 0.7038, and independent interlaboratory length-density measurements demonstrated high concordance with an *R*^2^ of 0.85. The stereology-based approach also demonstrated improved reproducibility compared with manual European consensus grading, with 92% precision compared with interpathologist concordance of 54.2%–65.2% and intrapathologist concordance of 60.9%.

The current study extends this prior work by applying automated fibrosis quantification across WSIs through automated reticulin-grid intersection counting in retained marrow tiles. Tile-level features were extracted using a vision transformer model and aggregated with multiple instance learning (MIL) to predict slide-level quantitative fibrosis scores. We further evaluated random and systematic tile-sampling strategies to reduce computational burden while preserving predictive performance. In addition, fat-excluded fibrosis scores were developed to investigate the impact of bone marrow adiposity on fibrosis assessment. Cluster-based interpretability analyses were also performed to identify histological phenotypes associated with model-predicted fibrosis.

## Materials and methods

### Histology, special staining, and whole-slide imaging

Bone marrow core biopsy specimens were processed using standard clinical histopathology procedures. Core biopsy specimens were fixed, decalcified using an EDTA-based decalcification protocol, paraffin embedded, sectioned at 4 μm, and stained for reticulin using Gomori silver impregnation for marrow reticulin fiber visualization. Slides were digitized using Aperio Leica Biosystems GT450 scanners. They were scanned at 40× equivalent magnification with a pixel resolution of approximately 0.2636 μm/pixel. WSIs were stored as pyramidal RGB SVS files and used for downstream automated reticulin detection, tissue-compartment segmentation, and fibrosis quantification.

### Study design and whole-slide image processing pipeline

WSIs were processed using an automated computational pathology pipeline to quantify reticulin-associated marrow fibrosis (Fig. 1, Fig. 2, Fig. 3). The pipeline included tissue prefiltering, tile selection, SegFormer-based tissue compartment segmentation, UNI2-h feature extraction, reticulin object detection, and Merz-grid stereological fibrosis quantification. The resulting slide-level Merz-derived fibrosis measurements were used as continuous reference labels for downstream MIL model development. Post hoc cluster-based analyses were performed to support model interpretability.

**Fig. 1:**
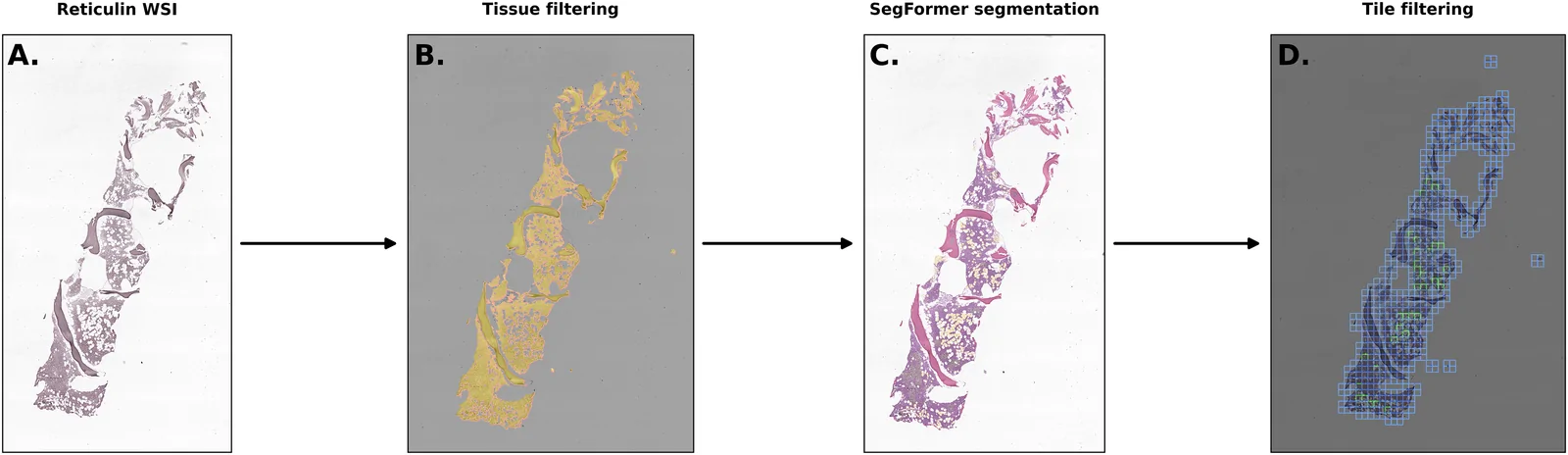
Methods workflow. Reticulin-stained whole-slide images were processed using an automated computational pathology pipeline for slide-level fibrosis quantification and prediction. Panel A demonstrates a whole-slide image before processing. Panel B illustrates how whole-slide images first underwent tissue prefiltering to identify candidate tissue-containing regions. Panel C shows SegFormer-based tissue compartment segmentation to classify background, trabeculae, cellular marrow, and fat. Panel D shows green and blue tiles, where green tiles passed predefined tissue-composition criteria were retained for downstream analysis. Retained tiles were used for reticulin object detection, Merz-grid fibrosis quantification, and UNI2-h feature extraction. (For interpretation of the references to color in this figure legend, the reader is referred to the web version of this article.)

**Fig. 2:**
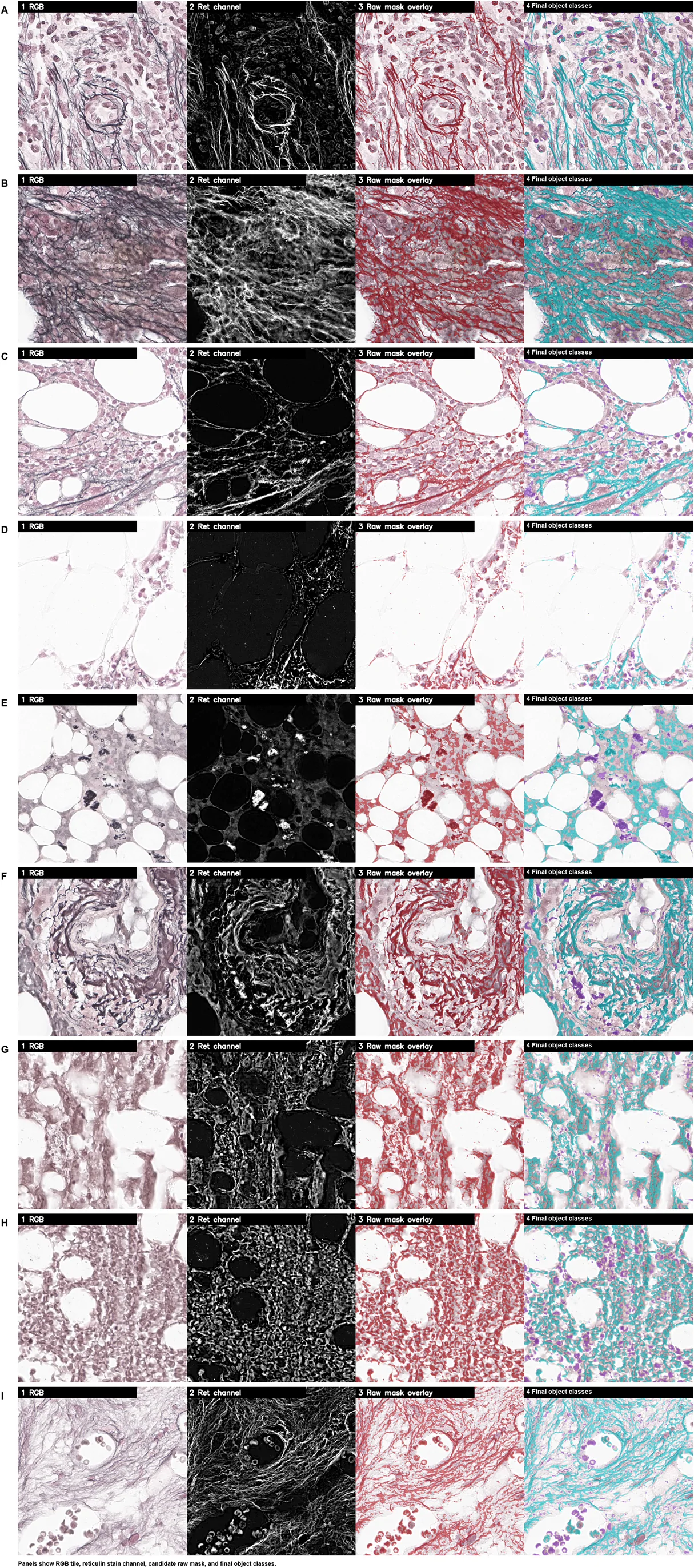
Reticulin object detection. A–I. Representative examples of reticulin detection, including correct detection, false-positive detection, and false-negative detection. From left to right, each row shows: (1) the original RGB region, (2) the reticulin grayscale channel used for Otsu thresholding, (3) the RGB reticulin mask after Otsu thresholding, and (4) final object classes after circularity-based filtering. Detection was most accurate in regions shown in panels A–D. False positives often occurred in intensely stained or folded regions, as illustrated in panels E, G, and H. False negatives occurred in densely fibrotic regions or areas with wispy reticulin networks, as illustrated in panels F and I.

**Fig. 3:**
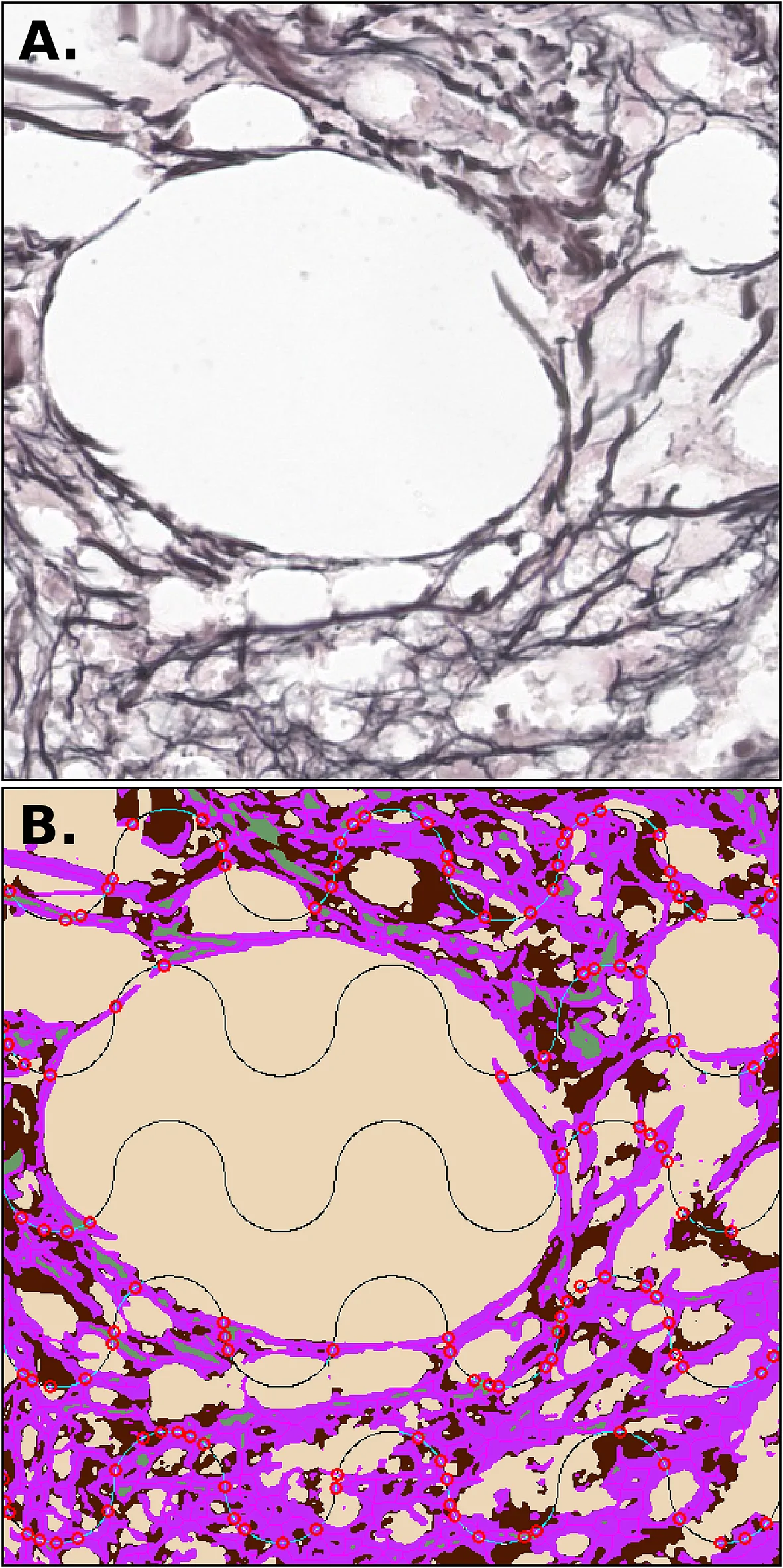
Merz fibrosis quantification. (A) Representative retained tile from a fibrotic marrow region after tissue filtering. (B) Merz-grid overlay applied to the same region. Magenta indicates reticulin, maroon indicates primary cellular marrow, green indicates objects initially classified as reticulin but excluded by circularity filtering and reassigned to cellular marrow for fibrosis computation, and tan indicates fat or background. Five Merz-grid lines are overlaid on the tile. Blue line segments indicate grid length passing through cellular marrow, whereas black line segments indicate grid length passing through fat or background. Grid-reticulin intersections are marked by red circles. Intersections were summed across each slide and divided by total eligible grid length, either including all eligible grid length or excluding fat-associated grid length from the denominator. (For interpretation of the references to color in this figure legend, the reader is referred to the web version of this article.)

### Tissue filtering and tile selection

WSIs were divided into non-overlapping 512 × 512-pixel tiles. Before semantic segmentation, candidate tiles were screened using a low-resolution tissue prefilter and a full-resolution blank-tile filter to reduce unnecessary processing of background-dominant regions (Fig. 1B).

For the low-resolution prefilter, slide thumbnails were generated. Tissue candidate regions were identified using saturation and dark-gray intensity thresholds, followed by morphological dilation and removal of small artifact components, including dust, slide-label interfaces, and isolated non-tissue regions. Tiles were retained for further evaluation when they exceeded a predefined low-resolution tissue-fraction threshold, with a margin applied to avoid excluding borderline tissue-containing regions.

### SegFormer-based tissue compartment segmentation

Candidate tiles that passed the tissue prefilters were analyzed using a four-class SegFormer-B4 semantic segmentation model developed for this study (Fig. 1C).^7^ The model used the original SegFormer-B4 architecture and assigned each pixel to one of four classes: background, trabeculae, cellular marrow, or fat. Background was treated as a non-target catch-all class that included red blood cells, crush artifact, and other non-target regions, whereas model development and validation focused on the three target tissue compartments: trabeculae, cellular marrow, and fat.

Model development used 58 manually labeled 512 × 512-pixel image/mask pairs from slides representative of the study cohort. Pairs were split at the slide level for training to prevent leakage during training and validation. Segmentation performance was evaluated using Dice similarity coefficients. The best development model achieved a target-class mean Dice of 0.92, with class-specific Dice scores of 0.96 for trabeculae, 0.92 for cellular marrow, and 0.87 for fat. Segmentation outputs were used to calculate tile-level tissue compartment fractions and guide tile retention decisions.

Tiles were retained when cellular marrow and fat comprised at least 95% of pixels, trabecular bone was minimal, and the combined fat/background fraction was <80%. This restricted downstream analyses to analyzable marrow regions. Tiles were also dropped when the combined fat and background fraction was ≥80%, thereby excluding regions dominated by adipose tissue, background, or both. The resulting retained tile table restricted downstream UNI2-h feature extraction, reticulin object detection, and Merz-grid quantification to analyzable marrow regions.

### Reticulin object detection

For each slide, retained 512 × 512 pixel regions were analyzed to identify reticulin-positive structures (Fig. 1D and Fig. 2). Reticulin detection was performed after color deconvolution, which generated a reticulin stain channel for each tile. Otsu thresholding was used to generate an adaptive candidate foreground mask on the reticulin stain channel, with a fixed minimum threshold of 0.20 to reduce detection of weak background signal.^8^ The resulting candidate mask was refined with light morphological opening and closing.

Connected objects were then extracted from the candidate mask. Because occasional cells and compact non-specific structures were included in the initial reticulin mask, circularity filtering was used to exclude round objects. Objects with circularity ≥0.25 were excluded as compact non-reticulin objects, whereas elongated or branching lower-circularity objects were retained as reticulin (Fig. 2). Parameter settings were selected through iterative visual quality-control review of randomly sampled tiles across slides. Final reticulin masks and object classifications were manually reviewed for segmentation quality before downstream fibrosis quantification. Detected reticulin objects were exported as GeoJSON annotations for downstream Merz-grid quantification.

### Merz-grid fibrosis quantification

Reticulin-positive objects were quantified using a Merz-grid–based stereological approach adapted from a prior computational study.^6^ Reticulin object GeoJSON annotations generated during object detection were overlaid with a Merz grid to automatically compute tile-level reticulin-grid intersection measurements and slide-level fibrosis summaries (Fig. 3).

Fibrosis scores were calculated as the total number of reticulin-grid intersections divided by the total eligible Merz-grid length across all retained tiles from a given slide. Two denominator definitions were used. For the all-length fibrosis score, the denominator included all eligible Merz-grid length. For the fat-excluded fibrosis score, the denominator was reduced by subtracting eligible grid length that passed through fat-containing regions. Both scores were multiplied by 1000, such that final values represented the number of reticulin-grid intersections per 1000 eligible grid pixels (Eqs. 1 and 2). Thus, the all-length score reflects reticulin intersections relative to all eligible marrow-core grid length, whereas the fat-excluded score reflects reticulin intersections relative to the non-fat eligible marrow compartment. These continuous slide-level measurements served as approximations of reticulin linear length density.

Automated Merz and vision transformer fibrosis scores were validated against the original Merz-grid stereology methodology.^6^ Matched reticulin-stained bone marrow core biopsy slide images (*n* = 602) were compared with manual Merz-grid measurements from the prior protocol, in which 10 randomly selected 200 × 200 μm regions were assessed per slide, reticulin-Merz-grid intersections were manually counted, and counts were averaged to generate a slide-level mean. This prior stereology protocol was previously validated against European consensus MF-0 to MF-3 grading, with linear regression *R*^2^ = 0.7038 versus average pathologist score.^6^ Morphological grading was performed in an independent fashion on each case by four experienced hematopathologists across two sites for interpathologist concordance. For intrapathologist concordance, two hematopathologists reviewed the same set of cases twice with a 1-month washout period. The pathologists were blinded for the clinical characterization of the patients from whom the samples had been taken. Correlation metrics were computed to compare the automated all-length and fat-excluded fibrosis scores with the manual Merz-grid measurements. To assess whether disagreement varied by fibrosis severity, manual Merz-grid scores were stratified into five fibrosis ranges from low to high, and automated-minus-manual residuals were summarized within each range.

### UNI2-h histology feature extraction

Retained tiles were processed using the frozen UNI2-h histology foundation model and its associated image transformation pipeline.^9^ For each retained 512 × 512-pixel tile, the corresponding WSI region was encoded into a 1536-dimensional feature vector. Tile embeddings, spatial coordinates, slide identifiers, and associated metadata were stored for downstream modeling.

### Slide-level dataset assembly for MIL modeling

Slide-level Merz fibrosis summaries were linked to corresponding UNI2-h embedding files. Slides were excluded from modeling if they contained fewer than 10 retained tiles (approximately, 1% of slides).

Two slide-level fibrosis targets were modeled separately: the all-length fibrosis score and the fat-excluded fibrosis score. The respective fibrosis scores were used as continuous regression endpoints for MIL model development.

### Train, validation, and locked test splitting

For each fibrosis target, slides were split at the patient level to reduce patient-level data leakage. Patient-level mean fibrosis scores were divided into quantile-based stratification bins, and patients were assigned to training, validation, and locked test sets using stratified splitting when possible (Table 1). Fifteen percent of patients were reserved as a locked test set, and 15% were reserved for validation.

**Table 1:** Cohort characteristics and fibrosis score distribution.

Split	Slides, No.^a^	Patients, No.	Tiles, No.	Tiles per slide, median	Tiles per slide, mean	Tiles per slide, range	Fibrosis score, mean ± SD	Fibrosis score, median	Fibrosis score, IQR^b^	Fibrosis score, range
Overall usable cohort	643	391	131,965	146.0	205.2	10–1913	28.79 ± 6.84	28.68	24.54–32.50	6.67–58.56
Training	451	273	90,822	139.0	201.4	10–1913	28.68 ± 6.57	28.31	24.57–32.55	6.67–58.56
Validation	96	59	16,869	148.5	175.7	11–733	29.42 ± 8.01	28.64	23.93–32.80	13.21–55.79
Locked test	96	59	24,274	172.5	252.9	19–1865	28.67 ± 6.79	28.98	24.86–32.06	12.63–43.24

Cohort characteristics are shown overall and by model split. Tiles represent retained 512 × 512-pixel regions used for downstream modeling. Fibrosis scores were computed at the slide level using automated Merz-grid quantification. Fibrosis scores represent reticulin-grid intersections per 1000 eligible grid-line pixels.

a No., number.

b IQR, interquartile range; SD, standard deviation. Patients were stratified by fibrosis severity and assigned to training, validation, or locked test cohorts with patient-level grouping to prevent leakage across splits.

### Multiple instance learning model development

A gated attention MIL regression model was trained to predict slide-level fibrosis scores from bags of tile-level UNI2-h embeddings.^10^ Specifically, MIL models were trained to predict the automated Merz-derived fibrosis scores rather than pathologist-assigned fibrosis grades. Each slide was represented as a bag of retained tile embeddings. During training and evaluation, random and systematic-random sampling strategies were evaluated using tile budgets of 8, 16, 32, 64, and 128 tiles per slide. During systematic sampling, tiles were ordered spatially using their x–y coordinates, and a random offset was used to select approximately evenly spaced tiles across the slide. Slides with fewer than the set number of tiles to sample used all available tiles.

Tile embeddings were normalized, projected through a fully connected layer with nonlinear activation and dropout, and aggregated using gated attention pooling. The resulting slide-level representation was passed to a regression head to predict the scaled fibrosis score.

### Model training and validation

Models were trained separately for the all-length and fat-excluded fibrosis targets. Target values were standardized using the mean and standard deviation of the training set during model development. Training used the AdamW optimizer.^11^ Huber loss was used as the regression loss function.^12^

Models were trained for up to 50 epochs with early stopping after 8 epochs without improvement in validation root mean squared error (RMSE). At each epoch, validation performance was estimated using five repeated systematic-random resamples per slide. Predictions were averaged at the slide level across resamples, and the model checkpoint with the lowest validation RMSE was selected as the best development model. Performance was summarized using RMSE, mean absolute error (MAE), *R*^2^, Pearson correlation, and Spearman correlation (Fig. 4).

**Fig. 4:**
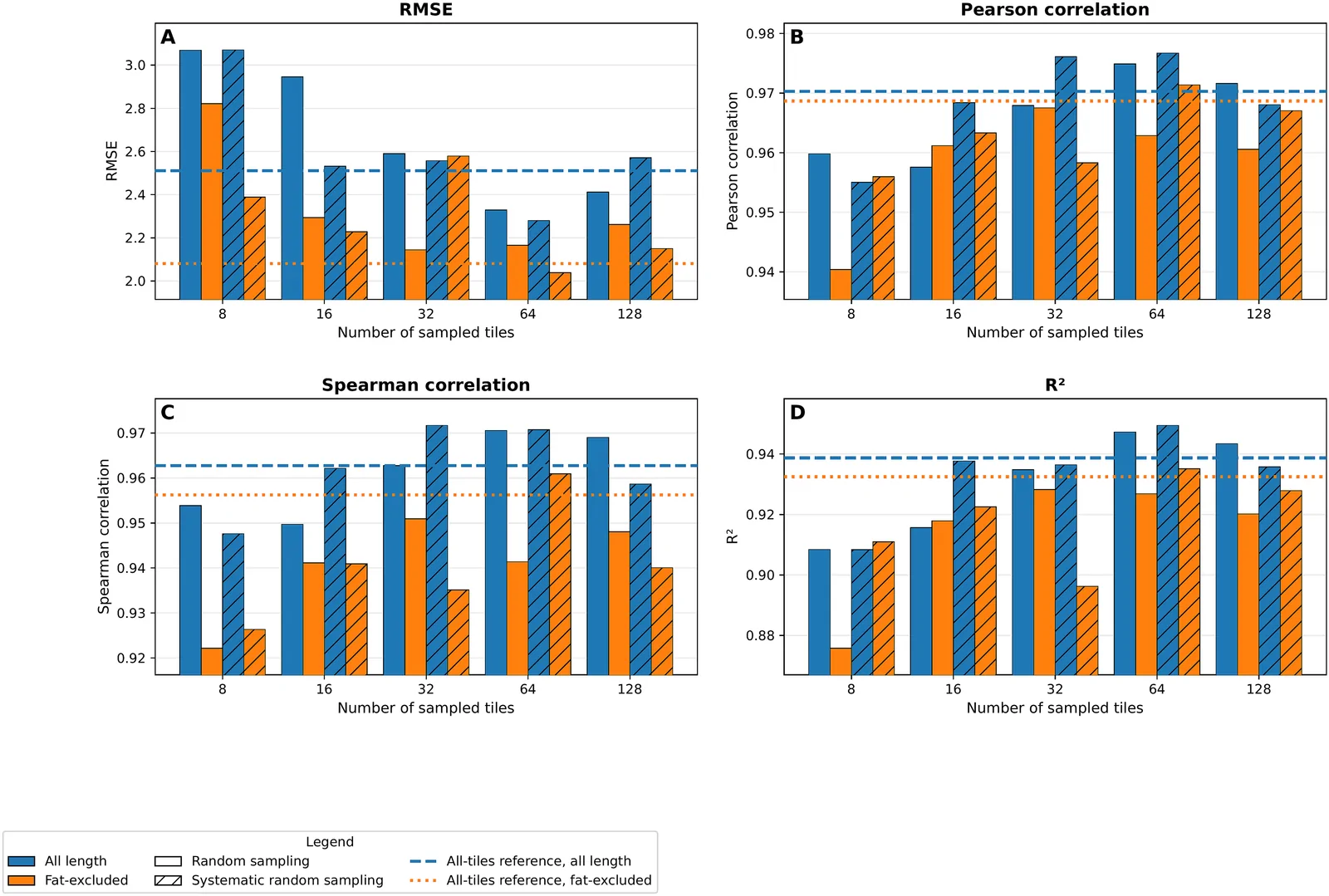
Validation metrics by sampling strategy. Validation performance is shown for the all-length and fat-excluded fibrosis prediction models across random and systematic-random tile sampling strategies. Tile budgets of 8, 16, 32, 64, and 128 tiles per slide were evaluated, with all retained tiles shown as a dashed horizontal reference line. (A) Root mean squared error (RMSE). (B) Pearson correlation. (C) Spearman correlation. (D) R^2^. Systematic-random sampling generally outperformed random sampling, and systematic-random sampling of 64 tiles per slide was selected as the final configuration for both fibrosis targets.

### Final model training and locked test evaluation

The final model configuration was selected based on the best training and validation performance. Before locked test evaluation, the selected configuration was retrained on the combined training and validation cohorts to maximize the number of cases available for final model fitting. The final model was then applied to the locked test cohort using the selected 64-tile systematic-random sampling strategy. Because systematic-random tile selection used a random offset, test-set inference was repeated using 50 tile-sampling resamples per slide. Predictions were averaged across resamples to obtain one final slide-level prediction for each case, after which locked test performance metrics were calculated. Test-set attention values were exported for downstream interpretability analysis.

### UMAP visualization of tile embeddings

To visualize the structure of the tile embedding space, Uniform Manifold Approximation and Projection (UMAP) was applied to standardized sampled training–validation embeddings used for cluster fitting.^13^ UMAP plots were colored by cluster assignment and tile attention to support qualitative review of cluster morphology and model-attention patterns (Fig. 6).

### Cluster-based and SHAP interpretability analysis

A post hoc cluster-based interpretability analysis was performed using the final trained MIL model. For this analysis, the final MIL model was applied to all available retained tiles from the combined training–validation cohort and the locked test cohort to obtain tile-level attention weights and slide-level model predictions. Tile clusters were fit only using training–validation embeddings to avoid test-set leakage.

Up to 300 tiles per slide were sampled from the training–validation cohort, with a maximum of 50,000 total tiles used for cluster fitting. Tile embeddings were standardized and clustered using MiniBatchKMeans to define a fixed set of shared tile phenotypes.^14^, ^16^ The resulting cluster model was then applied to both training–validation and locked-test tiles.

For each slide, cluster-level summary features were calculated using both the fraction of tiles assigned to each cluster and the summed MIL attention mass assigned to each cluster. These slide-level cluster features were used to train an ExtraTreesRegressor surrogate model to approximate final MIL model predictions.^15^ Five-fold cross-validation within the training–validation cohort was used to assess surrogate fidelity.

SHAP (Shapley Additive Explanations) values were calculated for the locked test-set using the fitted ExtraTrees surrogate model.^16^ Therefore, feature attributions reflect cluster-level contributions to the surrogate approximation of final MIL model predictions rather than direct explanations of individual pixels or raw neural network features. Mean absolute SHAP values were used to rank cluster-derived features according to their contribution to surrogate model predictions. Positive SHAP values indicated cluster features associated with higher predicted fibrosis scores, whereas negative SHAP values indicated cluster features associated with lower predicted fibrosis scores. Representative high-attention tiles were exported for each cluster to support morphological review of model-relevant tile phenotypes (Fig. 7, Panel C).

## Results

### Cohort characteristics

The study cohort comprised 643 WSIs from 391 patients, yielding 131,965 retained 512 × 512-pixel tiles for downstream model development and testing (Table 1). The median number of retained tiles per slide was 146.0, with a mean of 205.2 and a range of 10–1913 tiles per slide. The overall mean slide-level fibrosis score was 28.79 ± 6.84, with a median of 28.68, interquartile range of 24.54–32.50, and absolute range of 6.67–58.56.

Data were partitioned into training, validation, and locked test cohorts using patient-level stratification to reduce information leakage and preserve the distribution of fibrosis severity across experimental splits. The training set consisted of 451 slides from 273 patients and 90,822 tiles. The validation and locked test sets each contained 96 slides from 59 patients, comprising 16,869 and 24,274 tiles, respectively. Mean fibrosis scores remained consistent across the cohorts: training, 28.68 ± 6.57; validation, 29.42 ± 8.01; and locked test, 28.67 ± 6.79. Fibrosis score ranges were also overlapping across cohorts: training, 6.67–58.56; validation, 13.21–55.79; and locked test, 12.63–43.24. These findings indicate that the patient-level stratified sampling strategy preserved a broad spectrum of fibrosis severity across all experimental splits.

### Model selection and sampling strategy

Predictive models were independently optimized for two regression endpoints: the all-length fibrosis score and the fat-excluded normalized fibrosis score. A sampling sweep was performed to identify an efficient tile budget for slide-level prediction. Random and systematic-random sampling were evaluated at 8, 16, 32, 64, and 128 tiles per slide, with all available retained tiles included as a reference condition (Fig. 4).

Systematic-random sampling generally outperformed simple random sampling across the evaluated tile budgets. For the fat-excluded model, validation RMSE was 2.0 for systematic random 64, and 2.2 for random 64. For the all-length model, the difference was 2.28 and 2.33 (Fig. 4). A systematic-random budget of 64 tiles per slide was selected as the final configuration for both fibrosis targets, providing strong correlation and low prediction error while substantially reducing the number of tiles required for inference relative to all-tile processing.

For the all-length endpoint, the 64-tile systematic-random model achieved a validation RMSE of 2.28, MAE of 1.70, *R*^2^ of 0.95, Pearson *r* of 0.98, and Spearman ρ of 0.97. The non-fat–normalized model achieved a validation RMSE of 2.04, MAE of 1.61, *R*^2^ of 0.94, Pearson *r* of 0.97, and Spearman ρ of 0.96 (Table 2). Thus, the all-length model demonstrated slightly stronger validation correlation metrics, whereas the fat-excluded model demonstrated lower validation error.

**Table 2:** Validation and test performance of final slide-level fibrosis prediction models.

Fibrosis score target	Best model	Cohort	RMSE	MAE	*R*^2^	Pearson *r*	Spearman ρ
All-length	SR^a^ 64 tiles	Validation	2.28	1.70	0.95	0.98	0.97
All-length	SR^a^ 64 tiles	Locked test	2.97 (2.94–3.09)	2.06 (2.02–2.18)	0.86 (0.85–0.86)	0.93 (0.92–0.93)	0.92 (0.91–0.92)
Fat-excluded	SR 64 tiles	Validation	2.04	1.61	0.94	0.97	0.96
Fat-excluded	SR 64 tiles	Locked test	2.47 (2.35–2.62)	1.55 (1.54–1.64)	0.87 (0.85–0.88)	0.93 (0.92–0.94)	0.93 (0.92–0.95)

Values are reported for the final selected multiple instance learning models.

a Systematic-random 64 tiles indicates systematic-random sampling of 64 retained tiles per slide. RMSE indicates root mean squared error; MAE, mean absolute error; *R*^2^, coefficient of determination; Pearson *r*, Pearson correlation coefficient; and Spearman ρ, Spearman rank correlation coefficient. Fibrosis scores represent reticulin-grid intersections per 1000 eligible grid-line pixels. Locked test intervals represent the 2.5th to 97.5th percentile range across 50 tile-sampling resamples.

### Locked test performance

After model selection, final models were retrained on the combined training and validation cohorts and evaluated on the locked test cohort.

For the all-length model, resample-specific ranges were RMSE 2.94–3.09, MAE 2.02–2.18, *R*^2^ 0.850–0.864, Pearson *r* 0.923–0.931, and Spearman ρ 0.915–0.923. For the fat-excluded model, corresponding ranges were RMSE 2.35–2.62, MAE 1.54–1.64, *R*^2^ 0.851–0.881, Pearson *r* 0.924–0.940, and Spearman ρ 0.918–0.945 (Table 2). These results show that both models reproduced Merz-derived slide-level fibrosis quantification with strong held-out test performance. The non-fat–normalized model demonstrated lower absolute prediction error and slightly higher test-set *R*^2^, whereas correlation with the reference fibrosis score was comparable between the two targets. Although validation performance was higher than locked test performance, validation metrics were primarily used to guide model configuration. Locked test performance was assessed only after final model selection. The selected configuration was retrained on the combined training and validation cohorts, and locked test inference was repeated across 50 systematic-random tile-sampling resamples per slide to stabilize slide-level predictions before calculating final test metrics. Empirical uncertainty was summarized by recalculating test metrics for each of the 50 resamples and reporting the 2.5th–97.5th percentile range across resample-specific metrics.

### Validation against prior validated stereology method

The previously described stereology method was validated against the European consensus MF-0 to MF-3 grading scale, demonstrating correlation with average pathologist score by linear regression (*R*^2^ = 0.7038).^6^ Computer-generated stereology classification and subjective pathologist classification showed 56% concordance when individual MF-0 to MF-3 classes were compared, similar to reported interpathologist concordance of 54.2%–65.2% and intrapathologist concordance of 60.9%. However, the concordance reached 89% when 0/1 and 2/3 were grouped together, with a positive-predictive value of 96% and a negative-predictive value of 75%. The correlation between observers in two different labs showed high concordance (*R*^2^ = 0.85).

To validate the automated adaptation of this method, both automated fibrosis scores generated in the present study were compared with matched manual Merz-grid measurements (Fig. 5). Across 602 matched slide-level measurements, the all-length fibrosis score showed moderate correlation with manual stereology (Pearson *r* = 0.687; Spearman ρ = 0.665; OLS *R*^2^ = 0.472), with a MAE of 5.82 and RMSE of 7.63 relative to the manual slide-level mean. Predicted-versus-observed and residual analyses demonstrated that disagreement varied across the fibrosis spectrum. When stratified by manual Merz-grid fibrosis range, all-length error was lowest in the 10–20 range (MAE = 4.27) and highest in the >40 range (MAE = 10.75), with increasing underestimation at higher manual fibrosis scores. In contrast, the fat-excluded automated score showed weaker agreement with manual stereology (Pearson *r* = 0.362; Spearman ρ = 0.332; OLS *R*^2^ = 0.131), with higher overall error (MAE = 9.57; RMSE = 11.89). Fat-excluded error was lowest in the 30–40 range (MAE = 5.44) and highest in the ≤10 range (MAE = 18.59), with residual plots showing overestimation at low manual fibrosis scores and underestimation at higher manual fibrosis scores.

**Fig. 5:**
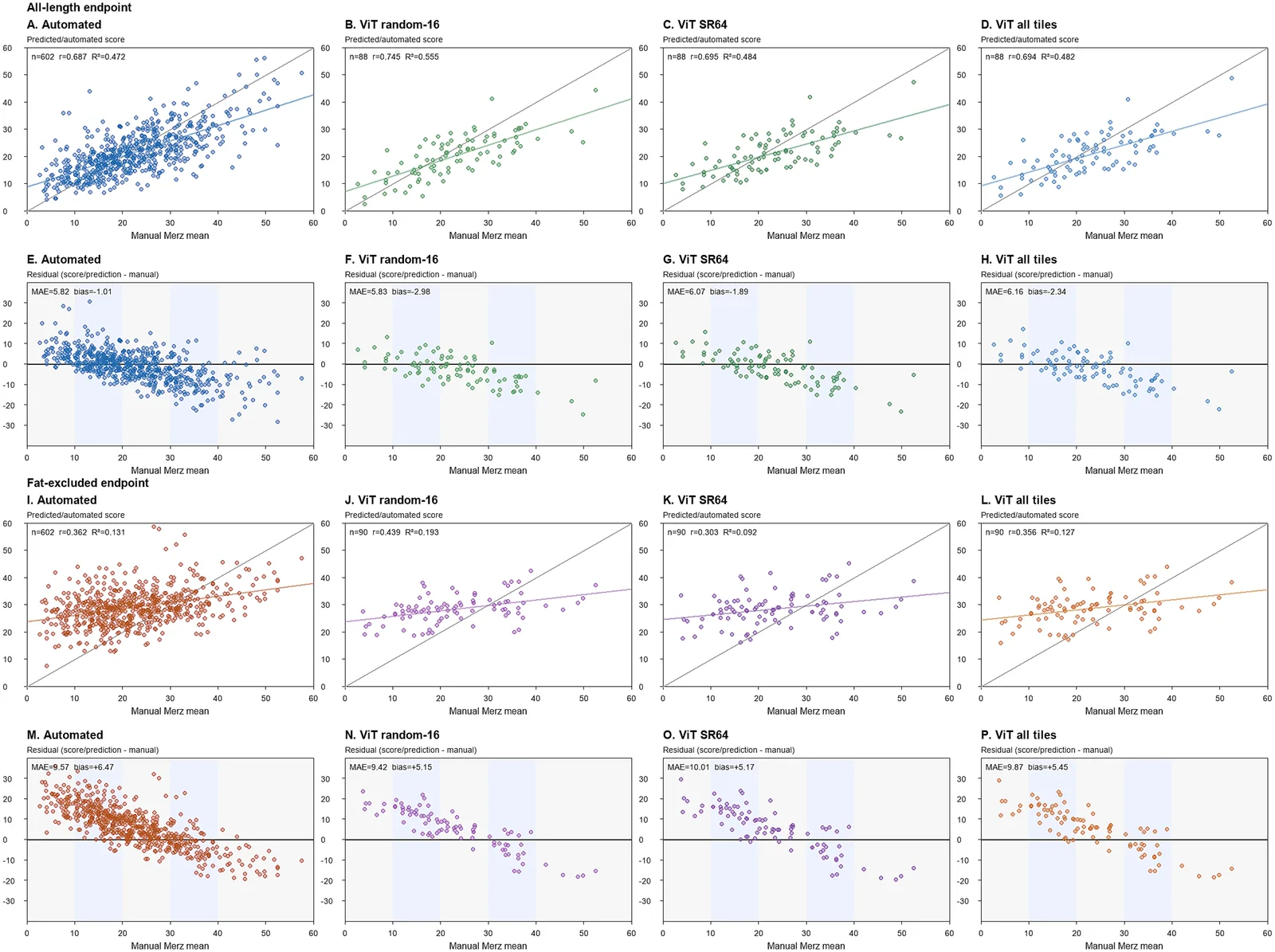
Automated and vision-transformer validation to manual Merz. Predicted-versus-observed and residual analyses comparing automated fibrosis scores and vision-transformer model predictions with matched manual Merz-grid stereology measurements. The all-length endpoint is shown in panels A–H, and the fat-excluded endpoint is shown in panels I–P. For each endpoint, columns show automated Merz-grid scoring, vision-transformer random 16-tile sampling, vision-transformer systematic-random 64-tile sampling, and vision-transformer all-tile inference. Predicted-versus-observed plots are shown above corresponding residual plots. Gray diagonal lines indicate identity, or perfect agreement; colored lines indicate ordinary least-squares regression fits. Residuals were calculated as automated score or model prediction minus manual Merz-grid mean. Shaded vertical bands indicate manual fibrosis-score ranges used for residual stratification. Abbreviations: ViT, vision-transformer; SR64, systematic-random sampling of 64 tiles per slide.

Vision-transformer (ViT) MIL model test predictions were then compared with the same manual Merz-grid measurements. For the all-length endpoint, the best locked-test correlation was observed using random sampling of 16 tiles per slide (*n* = 88; Pearson *r* = 0.745; Spearman ρ = 0.747; OLS *R*^2^ = 0.555; MAE = 5.83). This represented stronger correlation with manual Merz-grid stereology than the automated all-length score, although absolute error was similar. Correlations were similar across all-length sampling strategies, including random 8 tiles (Pearson *r* = 0.739; Spearman ρ = 0.747; OLS *R*^2^ = 0.546), random 32 tiles (Pearson *r* = 0.730; Spearman ρ = 0.726; OLS *R*^2^ = 0.533), systematic-random 64 tiles (Pearson *r* = 0.695; Spearman ρ = 0.698; OLS *R*^2^ = 0.484), systematic-random 128 tiles (Pearson *r* = 0.721; Spearman ρ = 0.722; OLS *R*^2^ = 0.520), and all tiles (Pearson *r* = 0.694; Spearman ρ = 0.675; OLS *R*^2^ = 0.482). Error analysis of the best all-length model showed that disagreement varied across manual fibrosis ranges, with lowest error in the 20–30 range (MAE = 4.21) and similar error in the 10–20 range (MAE = 4.22). Error was greatest in the >40 range (MAE = 16.29), where model predictions underestimated manual Merz-grid scores by a mean of 16.29. Residual plots showed a similar pattern of dynamic-range compression for all-length ViT predictions, with increasing underestimation at higher manual fibrosis scores.

For the fat-excluded ViT endpoint, locked-test correlations with manual stereology were weaker, with the best performance again observed using random 16 tiles per slide (*n* = 90; Pearson *r* = 0.439; Spearman ρ = 0.457; OLS *R*^2^ = 0.193; MAE = 9.42). Systematic-random 64-tile sampling showed lower correlation (Pearson *r* = 0.303; Spearman ρ = 0.271; OLS *R*^2^ = 0.092; MAE = 10.01), whereas all-tile inference showed slightly higher but still weak correlation (Pearson *r* = 0.356; Spearman ρ = 0.322; OLS *R*^2^ = 0.127; MAE = 9.87). Error analysis showed disagreement at both ends of the manual fibrosis spectrum. The fat-excluded model overestimated low-fibrosis cases, with highest error in the ≤10 range (MAE = 16.75; mean bias = +16.75) and underestimated the highest-fibrosis cases in the >40 range (MAE = 16.20; mean bias = −16.20). Agreement was best in the 30–40 range (MAE = 5.66). Together, these analyses indicate that all-length automated and ViT-derived scores more closely reproduce the prior manual Merz-grid method, whereas fat-excluded scores behave as a distinct compartment-normalized measurement.

### UMAP visualization of UNI2-h tile embeddings

UMAP visualization demonstrated structured organization of the UNI2-h tile embedding space (Fig. 6). Each point in the projection represented a sampled marrow tile, with color indicating cluster assignment from the shared clustering model. The separation of clusters within the UMAP projection suggests that UNI2-h embeddings captured morphologically distinct marrow tile phenotypes, even without explicit tile-level biological labels.

**Fig. 6:**
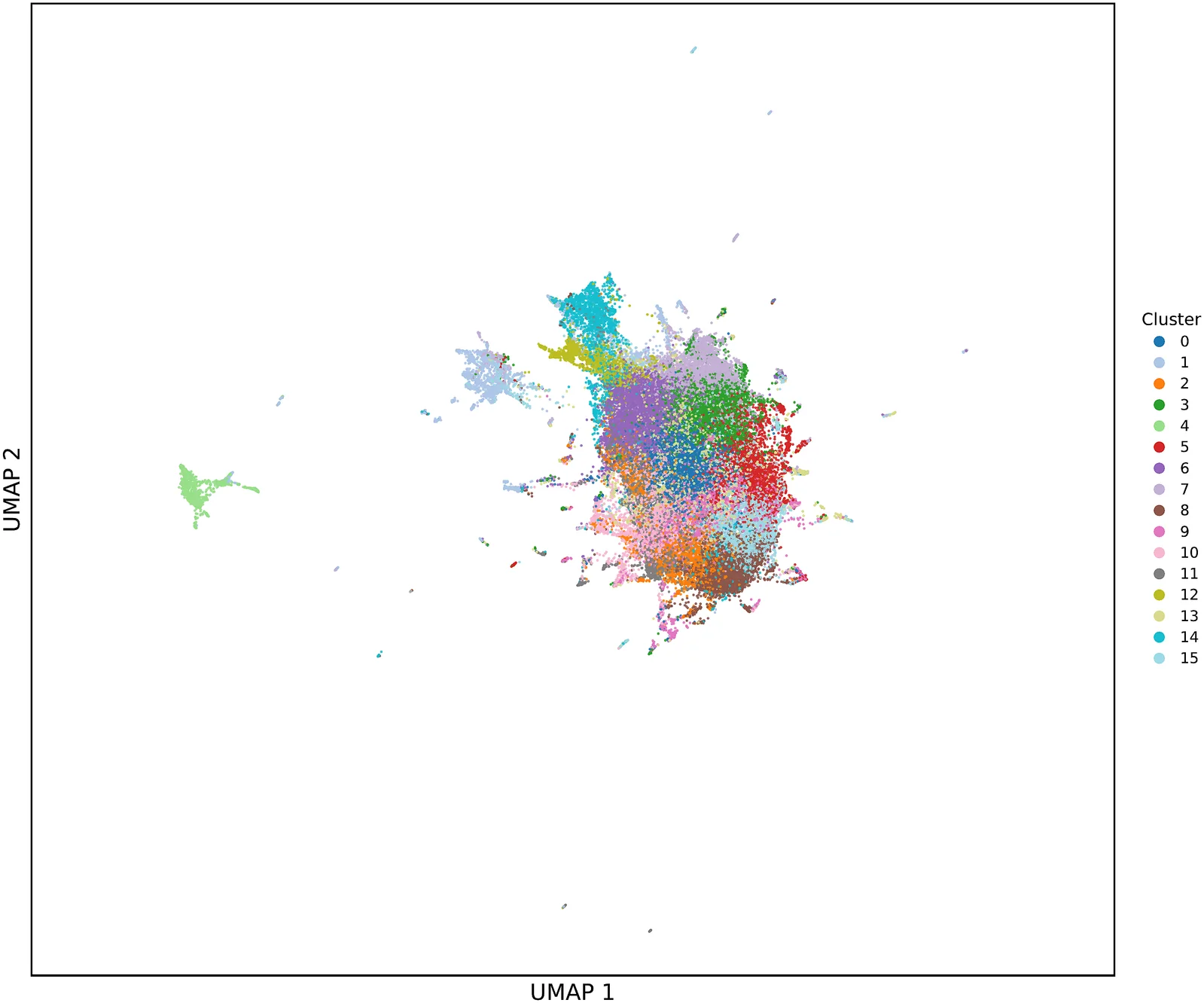
UMAP of UNI2-h tile embeddings from the shared training/validation cohort. Each point represents one sampled histology tile from the shared training–validation cohort. Tiles were embedded using UNI2-h and projected into two dimensions using UMAP. Colors indicate cluster membership assigned by the shared clustering model. UMAP 1 and UMAP 2 are projection coordinates and do not have direct biological units.

### Cluster-based SHAP interpretability

Post hoc cluster-based SHAP analysis was performed to characterize histological patterns associated with model predictions (Fig. 6, Fig. 7). A MiniBatchKMeans model trained on the development cohort assigned tiles to 16 shared phenotypic clusters (cluster 0–cluster 15), which were then applied to both all-length and fat-excluded models.

**Fig. 7:**
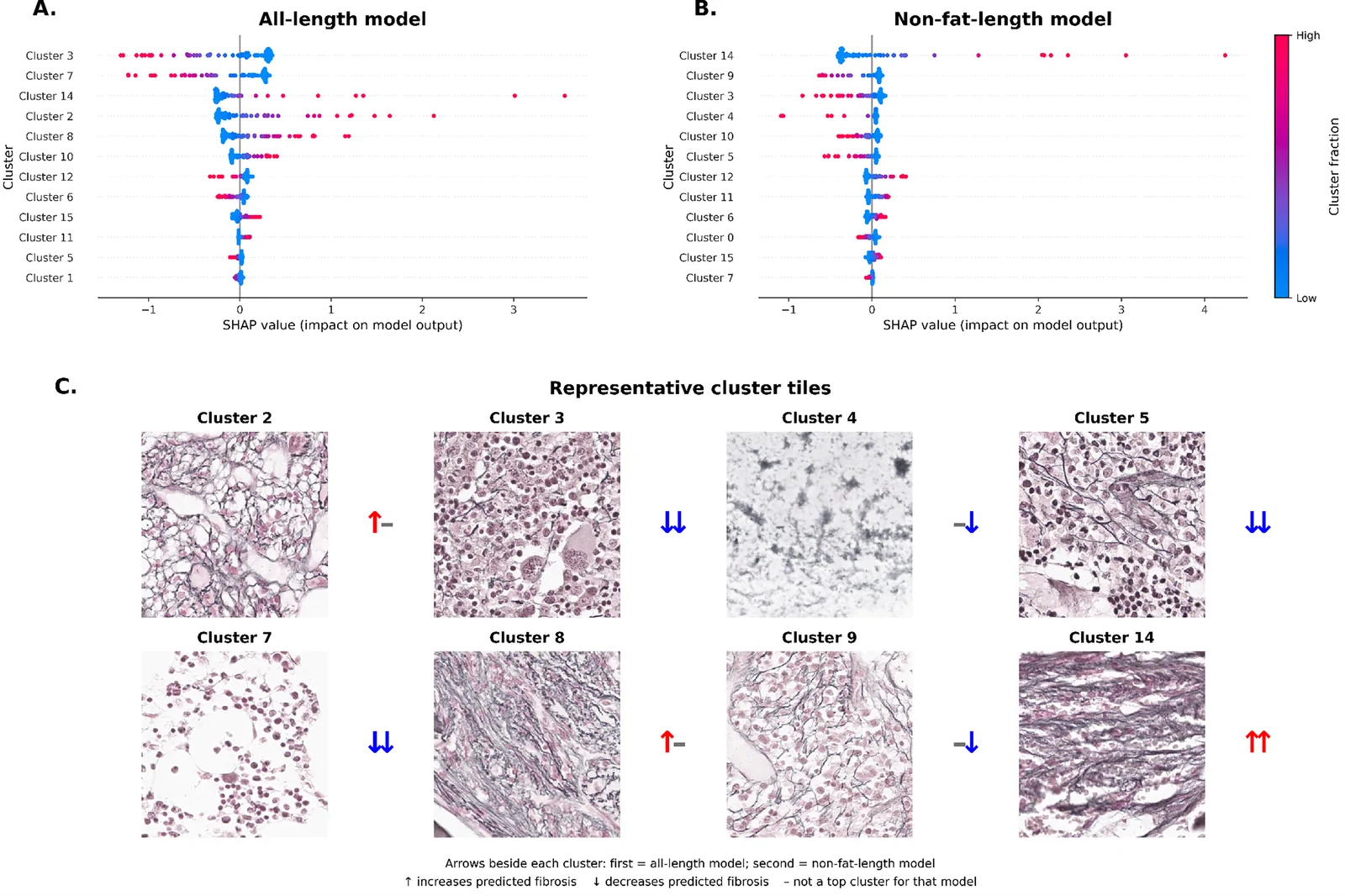
Cluster-level histological patterns associated with predicted reticulin fibrosis. Primary cluster-based SHAP analysis used MiniBatchKMeans to define a fixed set of tile phenotypes across the development cohort. 16 shared clusters were identified and applied to both the all-length and fat-excluded fibrosis models; the 12 clusters with the largest SHAP contributions are shown in Panels A–B. Panel C shows representative tile morphologies from high-attention clusters, with arrows indicating the direction of model attribution. Red arrows denote phenotypes associated with higher predicted fibrosis scores, whereas blue arrows denote phenotypes associated with lower predicted fibrosis scores. Left arrows indicate attribution for the all-length model, and right arrows indicate attribution for the fat-excluded model. A hyphen indicates that the morphology was not among the top contributing clusters for that model.

Slide-level cluster features, including cluster fractions and aggregated MIL attention mass, were used to train an ExtraTreesRegressor surrogate model to approximate MIL model outputs. SHAP values were calculated on the locked test cohort to quantify cluster-level contributions to the surrogate prediction of fibrosis.

The following cluster descriptions were assigned after systematic review of multiple representative tiles per cluster across multiple patients and slides. Cluster 0 grouped cellular tiles with loose reticulin networks. Cluster 1 grouped background and artifact. Cluster 2 contained cellular tiles with denser reticulin networks than Cluster 0. Cluster 3 grouped mixed cellular-adipocyte tiles with fine reticulin networks. Cluster 4 grouped slide background with silver deposits. Cluster 5 contained cellular tiles with minimal-to-moderate fibrosis. Cluster 6 contained adipocyte-rich tiles with moderately fibrotic networks. Cluster 7 contained adipocyte-rich tiles with minimal fibrosis. Cluster 8 contained cellular tiles with dense fibrotic networks and occasional vessels. Cluster 9 contained moderately fibrotic tiles in a cellular background. Cluster 10 contained cellular to mixed cellular-adipocyte tiles with dense reticulin networks and variable yellow-brown staining. Cluster 11 contained adipocyte-rich tiles with coarse reticulin strands and stromal septa. Cluster 12 grouped fat-predominant tiles with thick reticulin fibers along adipocyte borders. Cluster 13 contained mixed cellular-adipocyte marrow with moderate reticulin fibrosis. Cluster 14 contained stromal or fibrotic septal regions with elongated reticulin bundles. Cluster 15 contained hypercellular marrow tiles with variable reticulin staining and scattered dense fiber networks.

This interpretability framework identified morphological phenotypes associated with higher or lower predicted fibrosis scores. Clusters enriched for dense stromal or fibrotic-appearing patterns were associated with increased predicted fibrosis, whereas clusters representing adipocytic, low-cellularity, or low-fibrosis patterns were associated with lower predicted fibrosis. Review of representative high-attention tiles supported the biological plausibility of model-relevant cluster phenotypes (Fig. 7, Panel C). Because SHAP was applied to a surrogate model, these attributions should be interpreted as cluster-level explanations of the surrogate approximation of MIL predictions rather than direct explanations of individual image pixels.

## Discussion

### Summary of findings

In this study, we developed a whole-slide computational workflow for continuous quantification and prediction of reticulin fibrosis. The all-length endpoint showed closer correspondence with prior manual stereology than the fat-excluded endpoint, and robust slide-level prediction was maintained using a limited systematic-random tile sample. Error analysis identified underestimation at the upper end of the fibrosis range, whereas cluster-based analyses provided plausible morphological context for model interpretation.

### Methods validation

We previously showed that a stereology-based method to score fibrosis is comparable to conventional pathologist scoring (MF 0–MF 3). Additionally, we showed that this method is generalizable across two labs.^6^ Building on this approach, the current study automated tissue selection, reticulin object detection, Merz-grid intersection counting, and slide-level fibrosis summarization across WSIs (Fig. 1, Fig. 3). These continuous fibrosis measurements were then used as reference labels for attention-based MIL models using UNI2-h tile embeddings. We used ViT models as the final endpoint over automated counting in our study to improve reproducibility and simplify future computational analysis.

Validation in this study was completed by comparing prediction models and automated counter scores to our prior validated stereology approach. Our automated reticulin-grid intersection counter showed agreement with the previously described manual Merz-grid stereology method. Performance was stronger for the automated all-length fibrosis score (Pearson *r* = 0.687) than for the automated fat-excluded score (Pearson *r* = 0.362). The all-length MIL model similarly correlated stronger than fat-excluded (*r* = 0.745 versus 0.439) to the original method. Overall, MIL models showed stronger correlation than automated all-length counting on validation, although absolute error was similar. These findings suggest that MIL models capture fibrosis patterns more reliably than direct object detection.

Validation error analysis suggested that disagreement was not uniform for methods across the fibrosis spectrum. For the automated all-length score, error was lowest in slides with manual Merz-grid scores between 10 and 20 and highest in slides with scores >40, with a tendency toward underestimation in highly fibrotic cases. The all-length ViT model showed a similar pattern, with greatest error in the >40 range and underestimation of highly fibrotic cases. This pattern suggests partial compression of automated measurements or model predictions when compared with manually scored regions. This also suggests an area of improvement during model training. The fat-excluded score, and its corresponding ViT model, showed greater disagreement at the low end of the manual fibrosis range, consistent with inflation of scores in less cellular or more adipocytic marrows when adipose-containing grid length is removed from the denominator.

Overall, validation analysis indicates that the all-length score and prediction model agree with the prior validated method. Compared with fat-excluded, all-length is more directly interpretable within the context of the prior stereology method's validation. The fat-excluded score represents a distinct non-fat–compartment–normalized measurement that is not readily clinically interpretable.

### Tile budget

Strong slide-level prediction could be achieved using a limited tile budget. Systematic-random sampling of 64 tiles per slide provided robust validation and test performance for both fibrosis targets (Fig. 4; Table 2), suggesting that efficient sampling can substantially reduce computational overhead without requiring exhaustive all-tile inference. Additionally, validation of the vision transformer models against prior manual stereology demonstrated that the strongest correlation peaked during random sampling of 16 tiles for both endpoints, although did not greatly differ from sampling all tiles. This has practical relevance for future deployment because computational efficiency is an important consideration for large-scale WSI analysis.

### Vision-transformer model cluster interpretability

The interpretability analysis further supported the biological plausibility of the model. UMAP visualization showed structured organization of the UNI2-h embedding space (Fig. 6), and cluster-based SHAP analysis linked specific tile phenotypes to predicted fibrosis burden (Fig. 7, Fig. 8). Importantly, SHAP values were calculated from an ExtraTrees surrogate model trained on cluster-level features, so these attributions should be interpreted as phenotype-level explanations of the surrogate approximation of the MIL models rather than direct explanations of the neural network or individual image pixels. Representative high-attention tiles from influential clusters provided additional morphological context for model-relevant phenotypes (Fig. 7, Panel C, Fig. 8).

**Fig. 8:**
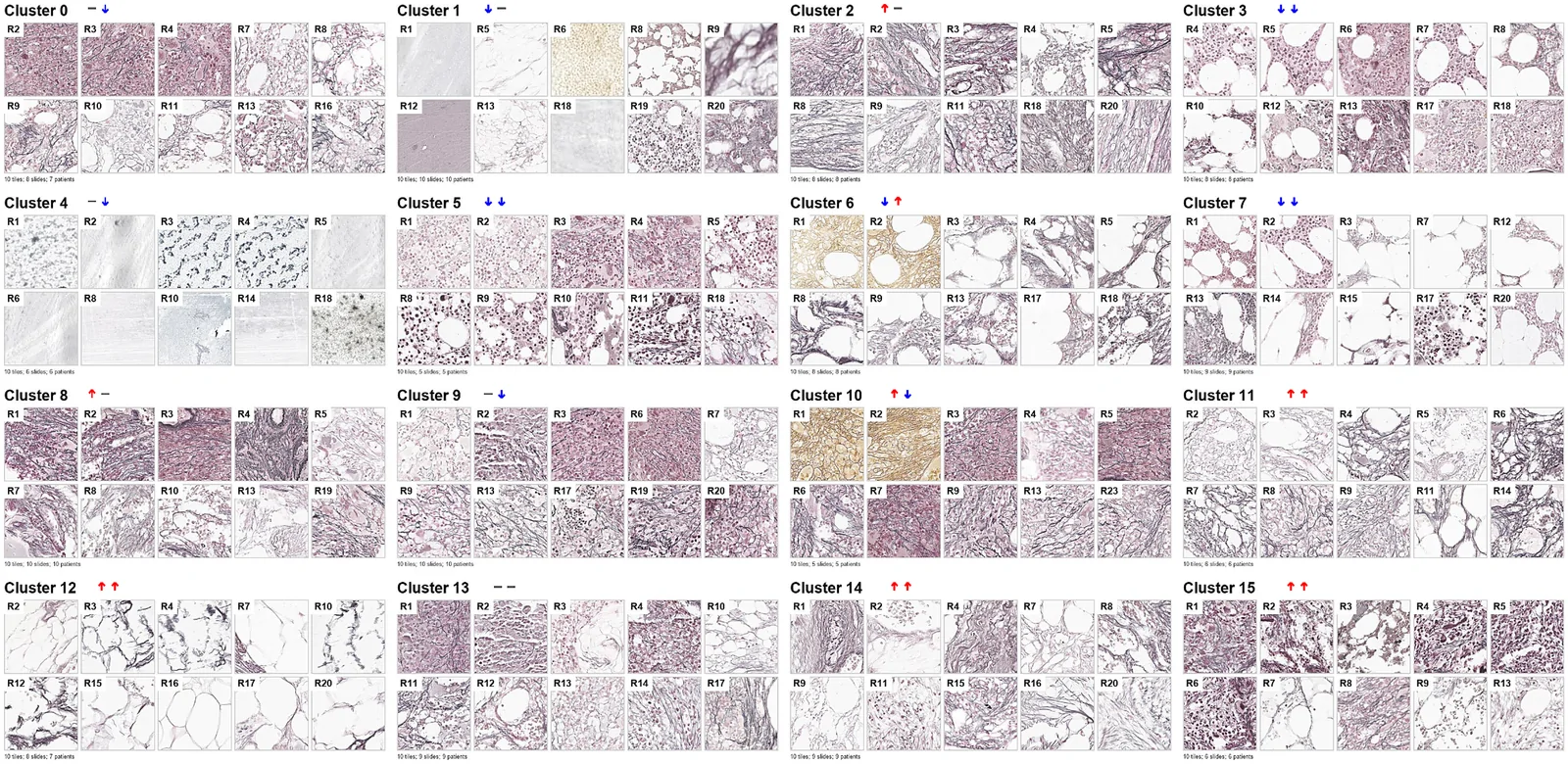
Cluster atlas with SHAP directional arrows. Examples of the 16 embedding clusters (clusters 0–15) used for post hoc vision-transformer model interpretability analysis. For each cluster, 10 representative high-attention tiles are shown. Tiles were preferentially selected from distinct slides and patients to better reflect cohort-level cluster morphology. These clusters summarize recurrent image patterns learned by the model and are intended as exploratory interpretability aids rather than validated histopathological classes. SHAP-based directional markers next to each cluster title indicate whether the cluster was associated with increased or decreased predicted fibrosis score for the all-length and fat-excluded endpoints, respectively. Numerical SHAP vectors are shown in Fig. 7.

### In the context of literature

The findings in our study extend a growing body of computational pathology work in MPN-associated marrow fibrosis. Ryou et al. introduced the continuous indexing of fibrosis framework, which used pairwise image comparisons to assign reticulin-stained marrow regions a continuous fibrosis score and achieved high pairwise-ranking accuracy.^1^ The same group later extended this framework to H&E-stained sections, supporting the feasibility of reticulin-free fibrosis estimation.^2^ Other recent approaches have focused on automated reticulin fibrosis grading or broader quantitative MPN bone marrow analysis, including an InceptionV3-based whole-slide fibrosis grading model by D'Abbronzo et al. and a U2-Net/ResNet-based quantitative marrow pathology platform by Yu et al.^3^, ^4^ In contrast to these approaches, the current study directly automated Merz-grid intersection counting across retained marrow tiles and used these continuous, grid-derived fibrosis measurements as slide-level reference labels for MIL-based prediction. Thus, the present workflow is complementary to prior grading, pairwise-ranking, and diagnostic classification approaches, while emphasizing scalable, quantitative, slide-level fibrosis measurement.

### Study limitations

This study has several limitations. First, the analysis was performed using an internal cohort, and external validation across institutions, scanners, staining protocols, and specimen-preparation workflows is needed. Second, although the Merz-grid–derived fibrosis score provides a continuous quantitative reference, it remains an adapted approximation of stereological length density rather than a direct three-dimensional measurement of reticulin fiber volume. Third, the interpretability analysis used cluster-level surrogate modeling, and therefore provides an indirect explanation of MIL model behavior. Finally, future studies should evaluate the relationship between continuous fibrosis predictions and clinical outcomes.

## Conclusion

This study evaluates the role of ViT models in continuous fibrosis prediction. Prior stereology-based fibrosis assessment demonstrated association with manual grading and improved reproducibility compared with pathologist scoring. In this study, the prior method was automated to count reticulin–grid intersection, and the resultant scores were used to train ViT models. Both all-length and fat-excluded models demonstrated strong locked test performance with limited tile sampling. Additionally, all-length models showed stronger correlation with the prior validated stereology method than the fat-excluded models. These findings support further evaluation of stereology-based ViT models for fibrosis quantification in clinical outcome studies for MPNs.

## Ethics statement

This study used deidentified WSIs. Formal institutional review board approval and informed consent were not required for the deidentified analysis.

## Funding

This research did not receive any specific grant from funding agencies in the public, commercial, or not-for-profit sectors.

## Declaration of competing interest

The authors declare that they have no known competing financial interests or personal relationships that could have appeared to influence the work reported in this article.

## Data Availability

Deidentified data supporting the findings of this study are available from the corresponding author upon reasonable request, subject to institutional and data-use requirements.

## Appendix A

(1) $$F_{\mathrm{all},s}=1000\times \frac{\sum_{t\in T_{s}}I_{\mathrm{st}}}{\sum_{t\in T_{s}}L_{\mathrm{st}}^{\mathrm{eligible}}}$$

The equation used for the fibrosis all-length target where *s* denotes the slide, *T*_*s*_ is the set of retained tiles from slide *s*, *I*_*st*_ is the number of reticulin-grid intersections from all retained tiles from slide *s*, and L_st_^eligible^ is the total eligible Merz grid length across *T*_*s*_.

**(**2) $$F_{\mathrm{fat}-\mathrm{excluded},s}=1000\times \frac{\sum_{t\in T_{s}}I_{\mathrm{st}}}{\sum_{t\in T_{s}}\left(L_{\mathrm{st}}^{\mathrm{eligible}}-L_{\mathrm{st}}^{\mathrm{fat}}\right)}$$

The equation used for the fibrosis fat-excluded target where *s* denotes the slide, *T*_*s*_ is the set of retained tiles from slide *s*, *I*_*st*_ is the number of reticulin-grid intersections from all retained tiles from slide *s*, and L_st_^eligible^ is the total eligible Merz grid length across *T*_*s*_, and *L*_*st*_^*fat*^ is the eligible grid length passing through fat-containing regions from *T*_*s*_.
